# XPD codon 312 and 751 polymorphisms, and AFB1 exposure, and hepatocellular carcinoma risk

**DOI:** 10.1186/1471-2407-9-400

**Published:** 2009-11-17

**Authors:** Xi Dai Long, Yun Ma, Yun Feng Zhou, Jin Guang Yao, Fu Zhi Ban, Yong Zhi Huang, Bing Cheng Huang

**Affiliations:** 1 Department of Pathology, Youjiang Medical College for Nationalities, Baise, Guangxi Zhuang Autonomous Region, PR China; 2 Department of Pathology, Guangxi Medical University, Nanning, Guangxi Zhuang Autonomous Region, PR China; 3 Department of Medicine, Affiliated Hospital of Youjiang Medical College for Nationalities, Baise, Guangxi Zhuang Autonomous Region, PR China; 4 Department of Medical Test, Affiliated Southwest Hospital of Youjiang Medical College for Nationalities, Baise, Guangxi Zhuang Autonomous Region, PR China

## Abstract

**Background:**

Genetic polymorphisms in DNA repair genes may influence individual variation in DNA repair capacity, which may be associated with risk of hepatocellular carcinoma (HCC) related to the exposure of aflatoxin B1 (AFB1). In this study, we have focused on the polymorphisms of xeroderma pigmentosum complementation group D (XPD) codon 312 and 751 (namely Asp312Asn and Lys751Gln), involved in nucleotide excision repair.

**Methods:**

We conducted a case-control study including 618 HCC cases and 712 controls to evaluate the associations between these two polymorphisms and HCC risk for Guangxi population by means of TaqMan-PCR and PCR-RFLP analysis.

**Results:**

We found that individuals featuring the XPD genotypes with codon 751 Gln alleles (namely XPD-LG or XPD-GG) were related to an elevated risk of HCC compared to those with the homozygote of XPD codon 751 Lys alleles [namely XPD-LL, adjusted odds ratios (ORs) were 1.75 and 2.47; 95% confidence interval (CIs) were 1.30-2.37 and 1.62-3.76, respectively]. A gender-specific role was evident that showed an higher risk for women (adjusted OR was 8.58 for XPD-GG) than for men (adjusted OR = 2.90 for XPD-GG). Interestingly, the interactive effects of this polymorphism and AFB1-exposure information showed the codon 751 Gln alleles increase the risk of HCC for individuals facing longer exposure years (*P*_interaction _= 0.011, OR = 0.85). For example, long-exposure-years (> 48 years) individuals who carried XDP-GG had an adjusted OR of 470.25, whereas long-exposure-years people with XDP-LL were at lower risk (adjusted OR = 149.12). However, we did not find that XPD codon 312 polymorphism was significantly associated with HCC risk.

**Conclusion:**

These findings suggest that XPD Lys751Gln polymorphism is an important modulator of AFB1 related-HCC development in Guangxi population.

## Background

Aflatoxin B1 (AFB1), the most common cause of high incidence rates of hepatocellular carcinoma (HCC) in Guangxi population [1,2], is an important chemical carcinogen and can induce AFB1-DNA adducts formation and cause DNA strand break, DNA base damage, and oxidative damage that may ultimately lead to cancer [3]. AFB1-induced DNA damage can be repaired by base excision repair (BER), strand break repair pathway, and nucleotide excision repair (NER) [4,5].

We have previously reported a relationship between HCC risk and allelic variations in the X-ray repair cross-complementing group 1 (XRCC1) and XRCC3 genes which are involved in BER and strand break repair pathway [6,7]. In this study, we focused on the xeroderma pigmentosum complementation group D (XPD) gene, which is one of the seven genetic complementation groups encoding for proteins involved in the NER pathway [5,8,9]. The two XPD polymorphic loci that have been of particular interest in molecular epidemiology studies are the Asp312Asn polymorphism in exon 10 and the Lys751Gln polymorphism in exon 23 [10]. Some studies have shown these two polymorphisms may be associated with decreased DNA repair capacity [11-13], increased frequency of p53 mutations [14-17], and increased tumor risk [13,18-30]. Therefore, we specifically conducted a hospital-based case-control study to examine whether these two polymorphisms modify the risk of HCC among Guangxi population from an AFB1 exposure area.

## Methods

### Study Subjects

HCC patients (n = 635) and control individuals (n = 712), who were residents of Guangxi Zhuang Autonomous Region from AFB1 exposure areas, were recruited from Affiliated Hospitals of the two main medical colleges in the Southwestern Guangxi (namely Guangxi Medical University and Youjiang Medical College for Nationalities) between January 2006 and August 2008. Cases and controls accepted to be enrolled in a proportion of 100%. The part of HCC patiens (n = 17) was excluded from this study because of the insufficient DNA samples. Thus, a total of 618 HCC cases, including 119 patients previously studied [6,31], were included in present analysis. Cases included in present study, representing for a significant portion of HCCs from Guangxi population, were identified through hepato surgery, hepato pathology, oncology, hepatology centres and through cancer registries, and confirmed by histopathological diagnosis in 100% of the HCC cases. During the same period, controls without clinical evidence of liver diseases (including 122 subjects previously studied [6]) were randomly selected from a pool of healthy volunteers that visited the general health check-up center of the same hospitals because of their routine scheduled physical exams supported by local governments.

To control the effects of confounders which were likely risk factors for Guangxiese HCC, controls were frequently matched to cases based on ethnicity (Han, Minority), sex, age (± 5 years), and hepatic B virus (HBV) and HCV infection. These having hepatitis B surface antigen (HBsAg)-positive and anti-HCV-positive in their peripheral serum were defined as groups infected HBV and HCV. With informed consent, the characteristic information of all study subjects, including sex, age, ethnicity, HBsAg and anti-HCV, were ascertained as described previously [7], at the same time 4 ml of peripheral blood was also collected.

The study protocol was been carried out in accordance with "Ethical Principles for Medical Research Involving Human Subjects" (World Medical Association Declaration Of Helsinki, 2004) and approved by Institutional review boards from Guangxi Cancer Institute, and Guangxi Government Medical Research Council.

### AFB1 Exposure

AFB1-exposure information included exposure-years and levels. In Guangxi, because food consumption types are relatively simple and limited corn, peanut, and rice, and AFB1 mainly contaminates these poorly stored food, especially, corn and peanut, the years of participants having food contaminated by AFB1 were defined as AFB1-exposure years for subjects. Cumulative AFB1 exposure-years were calculated by the following formula: exposure-years = (age - migration-years), where migration-years were defined as the years a person lived in non-exposure region of AFB1 (described in our previously published study [7]). To analyze, AFB1-exposure years were divided into three groups: short (< 40 years), medium (40 - 48 years), and long (> 48 years), according to the value of AFB1-exposure years, with two cutoff points of 40 and 48 years, the median exposure years among controls and cases.

In this study, we evaluated the AFB1-exposure levels by means of AFB1-DNA adducts levels of DNA samples from all subjects' peripheral blood leukocytes resulting from the fact that the DNA samples of liver tissue were not obtained from the controls. DNA was extracted from leukocyte samples by standard phenol-chloroform extraction, treated with 15 mM Na_2_CO_3 _and 30 mM NaHCO_3 _(pH 9.6) for 2 hrs to convert any N-7 adduct to AFB1-FAPy adducts, precipitated with 2.5 volumes of 95% ethanol, and then redissolved in 10 mM Tris-HCl (pH 7.0). The DNA samples were reprecipitated, dissolved in 1 × PBS, and denatured by boiling for 3 min and the AFB1-FAPy adducts were quantitated by competitive enzyme-linked immunosorbent assay (ELISA) using monoclonal antibody 6A10 (Novus Biologicals LLC, catalog # NB600-443) and 50 μg of DNA as described by Hsieh LL, *et al *[32]. The percent of inhibition was calculated by comparison with the nonmodified heat-denatured calf thymus DNA control (R&D Systems, Inc., catalog # 9600-5-D). DNA samples were assayed at 50 μg/well and quantitated relative to an imidazole ring-opened AFB1-DNA standard, which has a modification level of 4 adducts/10 nucleotides. Values about 10% inhibitions were corresponded to 0.25 μmol/mol DNA. Each sample was measured in triplicate on the three different assay dates and had a variability of less than 10%. Adduct levels were ascertained according to the average of three measures. The quality control for adduct assays was administered by blank and positive controls. In order to analyze, AFB1-DNA adduct levels were divided into three groups: low level (≤ 1.00 μmol/mol DNA), medium level (1.01 - 2.00 μmol/mol DNA), and high level (≥ 2.01 μmol/mol DNA), according to the value of AFB1-DNA adduct levels, with two cutoff points of 1.00 and 2.00 μmol/mol DNA, the average adducts levels among controls and cases.

### Genotyping

Gene polymorphism analysis of XPD Lys751Gln was typed by using the TaqMan-PCR on iCycler iQ™ real-time PCR detection system (iQ5, Bio-Rad). The primers were: 5'-AGT CAC CAG GAA CCG TTT ATG G-3' and 5'-TCT GTT CTC TGC AGG AGG ATC-3' (TaKaRa€). The probes were: 5'-HEX-CTC TAT CCT CTG CAG CG-TAMRA-3' and 5'-FAM-TAT CCT CTT CAG CGT CT-TAMRA-3'(TaKaRa). PCR reactions were run in a 25 μl final volume containing 1 × *Premix Ex TaqTM(*TaKaRa, catalog # DRR039A), 0.2 μM of each probe, 0.2 μM of each primer, and 50 - 100 ng of genomic DNA. Cycling conditions were 95°C for 30 s, and 50 cycles of 95°C for 10 s and 60°C for 1 min. Controls were included in each run and repeated genotyping of a random 10% subset yielded 100% identical genotypes. Data analysis for allele discrimination was performed with the iCycler iQ software. While Gene polymorphism analysis of XPD Asp312Asn was detected by using a previously published PCR-RFLP method [11].

### Statistical Analysis

All statistical analyses were done using the statistical package for social science (SPSS) version 11.5 (SPSS Institute, Chicago, IL). The Pearson χ^2 ^test was used to test for differences between the cases and the control subjects in the distribution of gender, age, ethnicity, HBV and HCV infection, AFB1-exposure information, and XPD genotypes. The odds ratio (OR) and the 95% confidence interval (CI) were calculated as an estimate of relative risk. Unconditional multivariate logistic regression was used to control for possible confounding by age, gender, ethnicity, HBV and HCV infection, and AFB1-exposure information, where appropriate, and when examining interactions between the polymorphisms and AFB1-exposure information (including exposure years and levels). Interaction was tested using a multiplicative interaction term included in the multivariate model. Joint effects between genotypes and exposure information were assessed by using the full regression model[33]. A *P*-value of < 0.05 was considered statistically significant.

## Results

### Demographic and etiologic characteristics for subjects

There were no significant differences for sex, age, ethnicity, HBsAg and anti-HCV status (*P *> 0.05; Table 1), which suggested HCC patients' data are comparable with controls'.

**Table 1:** Demographic and etiologic characteristics of HCC cases and controls

	Controls	Cases		
				
	No. (%)	No. (%)	χ^2^	Two-sided *P*
**Sex**			1.945	0.163
Male	540 (75.8)	448 (72.5)		
Female	172 (24.2)	170 (27.5)		
**Age(years)**^a^			2.672	0.263
< 35	86 (12.1)	58 (9.4)		
35-65	554 (77.8)	500 (80.9)		
> 65	72 (10.1)	60 (9.7)		
**Ethnicity**			0.891	0.345
Han	318 (44.7)	292 (47.2)		
Minority	394 (55.3)	326 (52.8)		
**HBV infection**			0.354	0.552
HBsAg (+)	508 (71.3)	450 (72.8)		
HBsAg (-)	204 (28.7)	168 (27.2)		
**HCV infection**			0.049	0.825
Anti-HCV (+)	128 (18.0)	114 (18.4)		
Anti-HCV (-)	584 (82.0)	504 (81.6)		

^a ^The mean ± S.E. ages were 49.31 ± 0.45 and 49.15 ± 0.43 for cases and controls, respectively.

### AFB1 exposure and HCC risk

Table 2 summarized AFB1-exposure information of all study population. We found that AFB1-exposure years (median) were longer among the cases (48 years) than the controls (40 years) and the risk of HCC gradually increased with the increasing exposure years (*P *< 0.01). We also found that the levels of AFB1-DNA adducts were positively associated with HCC risk (adjusted ORs were 2.11 and 6.23 for medium- and high-level adducts, respectively). These results were consistent with our previously published data [6,7]. Additionally, we also analyzed the association between AFB1-DNA adduct levels of peripheral blood leukocytes and those of the cancerous tissue of 119 HCC patients previously studied [31]. The results showed AFB1-DNA adduct levels of the HCC cancerous tissue were positively and linearly related to peripheral blood leukocytes' adduct levels (data not shown), which suggested that the levels of peripheral blood leukocytes' DNA adducts were representative of the tissues' DNA-adduct levels.

**Table 2:** AFB1 exposure and the risk of HCC

	Controls		HCCs				
					
AFB1 exposure	n	%	n	%	OR (95% CI)	Adjusted OR (95% CI)	*P*_trend_
AFB1-exposure years^a, b^							
Short	378	53.1	130	21.0	Reference	Reference	
Medium	197	27.7	196	31.7	2.89 (2.19-3.83)	9.08(6.07-13.57)^c^	< 0.001
Long	137	19.2	292	47.2	6.20 (4.66-8.24)	156.03(77.83-312.77)^c^	< 0.001
AFB1-DNA adducts levels^d, e^							
Low	380	53.4	153	24.8	Reference	Reference	
Medium	209	29.4	172	27.8	2.04(1.55-2.69)	2.11(1.54-2.90)^f^	< 0.001
High	123	17.3	293	47.4	5.92(4.46-7.84)	6.23(4.48-8.67)^f^	< 0.001

^a ^The Median for AFB1-exposure years were 48 years and 40 years for cases and controls, respectively.

^b ^AFB1-exposure years were: < 40 years for short exposure; 40 - 48 years for medium exposure; > 48 years for long exposure.

^c ^Adjusted for age, sex, ethnicity, HBsAg, anti-HCV, and AFB1-DNA adducts levels.

^d ^The mean ± S.E. level of AFB1-DNA adducts is 1.97 ± 0.05 and 1.04 ± 0.02 μmol/mol DNA for cases and controls, respectively.

^e ^Adduct levels were: ≤ 1.00 μmol/mol DNA for low level; 1.01 -- 2.00 μmol/mol DNA for medium; ≥ 2.01 μmol/mol DNA for high level.

^f ^Adjusted for age, sex, ethnicity, HBsAg, anti-HCV, and AFB1-exposure years.

### XPD polymorphisms and HCC risk

The genotypic distribution of XPD Asp312Asn and Lys751Gln for both cancer cases and controls were shown in Table 3. The frequencies of the codon 312 Asn allele and codon 751 Gln allele among cases (0.26 and 0.38, respectively) were higher than among controls (0.22 and 0.22, respectively). Genotypic distributions of these two loci in controls were in Hardy-Weinberg equilibrium [Chi-square (*P*-values) were 1.56 (0.458) and 2.704 (0.259) for codon 312 and 751 genotypes, respectively, data not shown]. However, logistic regression analyses showed that only the codon Lys751Gln polymorphism was significantly associated with the increasing risk of HCC. The adjusted OR for HCC for those individuals carrying the heterozygotes for codon 751 Lys and Gln allele (XPD-LG) compared with those exhibiting featuring the homozygote for Lys alleles (XPD-LL) was 1.75 (95% CI, 1.30 - 2.37), and the corresponding OR for those featuring the homozygote for Gln alleles (XPD-GG) was 2.47 (95% CI, 1.62 - 3.76), which showed the risk of HCC was associated with the number of codon 751 Gln alleles.

**Table 3:** The polymorphisms of XPD and the risk of HCC

	Controls		HCCs				
					
XPD genotype	n	%	n	%	OR (95% CI)	Adjusted OR (95% CI)^a^	*P*_trend_
XPD codon Asp312Asn							
DD^b^	453	63.6	364	58.9	Reference	Reference	
DN^b^	200	28.1	190	30.7	1.18(0.92-1.51)	1.12(0.83-1.51)	0.460
NN^b^	59	8.3	64	10.4	1.35(0.93-1.97)	0.89(0.56-1.41)	0.620
XPD codon Lys751Gln							
LL^c^	464	65.2	272	44.0	Reference	Reference	
LG^c^	187	26.3	222	35.9	2.03(1.58-2.59)	1.75(1.30-2.37)	< 0.001
GG^c^	61	8.6	124	20.1	3.47(2.47-4.88)	2.47(1.62-3.76)	< 0.001

^a ^Adjusted for age, sex, ethnicity, HBsAg, anti-HCV, AFB1-exposure years, and AFB1-DNA adduct levels.

^b ^DD, DN, and NN represented the homozygotes of XPD codon 312 Asp alleles, the heterozygotes of XPD codon 312 Asp and Asn allele, and the homozygotes of XPD codon 312 Asn alleles, respectively.

^c ^LL, LG, and GG represented the homozygotes of XPD codon 751 Lys alleles, the heterozygotes of XPD codon 751 Lys and Gln allele, and the homozygotes of XPD codon 751 Gln alleles, respectively.

### XPD codon 751 polymorphism and HCC risk stratified by HBV infection, HCV infection, and gender

To evaluate the modification of gender on the effect of risk genotypes (adjusted OR > 1) of XPD codon 751, we investigated the risk for HCC associated with the genetic polymorphism separately among female group and among male participants (Table 4). The results demonstrated that the genotypes with codon 751 Gln alleles were related to a higher risk of HCC for women than for men (*P*_interaction _< 0.01, OR = 0.43). When we compared the XPD-GG versus XPD-LL, the adjusted OR for women was 8.58 (95% CI, 3.28 - 22.46), whereas the adjusted OR for men was 2.90 (95% CI, 1.99 - 4.21).

**Table 4:** XPD codon 751 polymorphism and HCC risk stratified by HBV infection (HBsAg- negative and positive), HCV infection (anti-HCV- negative and positive), and gender (female and male)

		Controls		HCCs			
					
		n	%	n	%	OR (95% CI)	Adjusted OR (95% CI)
HBsAg^a^	XPD						
Negative	LL	153	75.0	86	51.2	Reference	Reference
	LG	32	15.7	56	33.3	3.11(1.87-5.18)	2.36(1.25-4.45)^b^
	GG	19	9.3	26	15.5	2.44(1.27-4.46)	1.42(0.60-3.36)^b^
	LG/GG	51	25.0	82	48.8	2.86(1.85-4.43)	2.01(1.16-3.51)^b^
Positive	LL	311	61.2	186	41.3	Reference	Reference
	LG	155	30.5	166	36.9	1.79(1.35-2.38)	1.52(1.08-2.16)^b^
	GG	42	8.3	98	21.8	3.90(2.60-5.85)	2.64(1.60-4.37)^b^
	LG/GG	197	38.8	264	58.7	2.24(1.73-2.90)	1.77(1.29-2.43)^b^
Anti-HCV^c^	XPD						
Negative	LL	387	66.3	223	44.2	Reference	Reference
	LG	150	25.7	185	36.7	2.14(1.63-2.81)	1.93(1.39-2.70)^d^
	GG	47	8.0	96	19.0	3.55(2.41-5.21)	2.93(1.82-4.72)^d^
	LG/GG	197	33.7	281	55.8	2.48(1.94-3.17)	2.17(1.60-2.94)^d^
Positive	LL	77	60.2	49	43.0	Reference	Reference
	LG	37	28.9	37	32.5	1.57(0.88-2.81)	1.28(0.70-2.34)^d^
	GG	14	10.9	28	24.6	3.14(1.51-6.55)	2.77(1.29-5.93)^d^
	LG/GG	51	39.8	65	57.0	2.00(1.20-3.34)	1.68(0.99-2.85)^d^
Sex^e^	XPD						
Female	LL	133	77.3	65	38.2	Reference	Reference
	LG	33	19.2	81	47.6	5.02(3.04-8.30)	5.64(3.33-9.56)^f^
	GG	6	3.5	24	14.1	8.19(3.19-21.01)	8.58(3.28-22.46)^f^
	LG/GG	39	22.7	105	61.8	5.51(3.44-8.83)	6.12(3.72-10.05)^f^
Male	LL	331	61.3	207	46.2	Reference	Reference
	LG	154	28.5	141	31.5	1.46(1.10-1.95)	1.50(1.12-2.01)^f^
	GG	55	10.2	100	22.3	2.91(2.00-4.22)	2.90(1.99-4.21)^f^
	LG/GG	209	38.7	241	53.8	1.84(1.43-2.38)	1.88(1.45-2.43)^f^

^a^Likelihood ration test for interaction of the stratified variable (HBsAg-negative and positive) and XPD codon 751 genotype was calculated as test for the heterogeneity of ORs across strata (interact term OR = 1.02, *P*_interaction _= 0.938).

^b^Adjusted for age, sex, ethnicity, anti-HCV, AFB1-exposure years, and AFB1-DNA adduct levels.

^c^Likelihood ration test for interaction of the stratified variable (anti-HCV-negative and positive) and XPD codon 751 genotype was calculated as test for the heterogeneity of ORs across strata (interact term OR = 0.87, *P*_interaction _= 0.492).

^d^Adjusted for age, sex, ethnicity, HBsAg, AFB1-exposure years, and AFB1-DNA adduct levels.

^e^Likelihood ration test for interaction of the stratified variable (female and male) and XPD codon 751 genotype was calculated as test for the heterogeneity of ORs across strata (interact term OR = 0.43, *P*_interaction _= 0.0001).

^f^Adjusted for age, ethnicity, HBsAg, anti-HCV, AFB1-exposure years, and AFB1-DNA adduct levels.

Because HBV and HCV infection are two important risk factors for HCC among the Guangxi population, we analyzed the modified effects between these two confounders and XPD codon 751 polymorphism on HCC risk (Table 4). Similar risk values for HCC were found among HBsAg-positive carriers and among HBsAg-negative participants (adjusted ORs were 1.77 and 2.01, respectively). Similar results were also found in the stratified analysis between among anti-HCV-positive carriers and among anti-HCV-negative group (adjusted OR was 1.68 and 2.17, respectively). Likelihood ration tests for interaction of the stratified variables [including HBsAg (negative and positive), and anti-HCV (negative and positive)] and XPD codon 751 genotypes were not statistically significant (*P*_interaction _was 0.938 and 0.492 for HBV and HCV infection, respectively).

### Joint effects of AFB1 exposure and XPD codon 751 polymorphism on HCC risk

To study the relationship between XPD codon 751 polymorphism and AFB1-exposure years in the risk for HCC, we analyzed the AFB1-exposure years-genotypes joint effect on HCC risk (Table 5). In this analysis, we used reference the lowest risk group: those who had the XPD-LL and short-term AFB1-exposure years. The results showed that increasing AFB1-exposure years consistently increased HCC risk, moreover, this effect was more pronounced among the XPD-LG or XPD-GG subjects. For example, individuals who featured long-term AFB1-exposure years and carried the XPD-LG had an adjusted OR of 290.55 (95% CI, 125.56 - 672.36), whereas those who had the XPD-GG had an adjusted OR of 470.25 (95% CI, 163.21-1354.96) relative to the reference group (Table 5). Interestingly, we found that the interaction between genotypes and AFB1-exposure years can modify the main effects of XPD codon 751 genotypes in the multiplicative model (*P*_interaction _= 0.011, OR = 0.85). Similar increasing-risk trend was also found in the joint effective analysis between XPD codon 751 genotypes and AFB1-DNA adducts levels for the risk of HCC, although the multiplicative interaction term was not statistically significant (*P*_interaction _= 0.525, OR = 1.04, Table 6).

**Table 5:** Joint effects of AFB1-exposure years and XPD codon 751 polymorphism on HCC risk

		Controls		HCCs			
					
AFB1-exposure years^a, b^	XPD genotypes	n	%	n	%	OR (95% CI)	Adjusted OR (95% CI)^c^
Short	LL	255	35.8	66	10.7	Reference	Reference
	LG	97	13.6	40	6.5	1.59(1.01-2.52)	1.30(0.77-2.20)
	GG	26	3.7	24	3.9	3.57(1.92-6.61)	3.70(1.80-7.59)
Medium	LL	124	17.4	78	12.6	2.43(1.64-3.60)	8.77(5.29-14.53)
	LG	47	6.6	70	11.3	5.75(3.64-9.10)	14.38 (7.26-28.48)
	GG	26	3.7	48	7.8	7.13(4.12-12.35)	18.52 (10.24-33.50)
Long	LL	85	11.9	128	20.7	5.82(3.96-8.55)	149.12(70.43-315.72)
	LG	43	6.0	112	18.1	10.06(6.46-15.68)	290.55(125.56-672.36)
	GG	9	1.3	52	8.4	22.32(10.47-47.62)	470.25(163.21-1354.96)

^a ^From the likelihood ratio test comparing the fit of the logistic model that included the main effects of AFB1-exposure years, genotypes and all potential confounders with a fully parameterized model containing the interaction terms of XPD codon 751 genotypes and AFB1-exposure years (interact term OR = 0.85, *P*_interaction _= 0.011).

^b ^AFB1-exposure years were: < 40 years for short exposure; 40 - 48 years for medium exposure; > 48 years for long exposure.

^c ^Adjusted for age, sex, ethnicity, HBsAg, anti-HCV, and AFB1-DNA adducts levels.

**Table 6:** Joint effects of AFB1-DNA adducts levels and XPD codon 751 polymorphism on HCC risk

		Controls		HCCs			
					
AFB1-DNA adducts levels^a, b^	XPD genotypes	n	%	n	%	OR (95% CI)	Adjusted OR (95% CI)^c^
Low	LL	257	36.1	64	10.4	Reference	Reference
	LG	94	13.2	68	11.0	2.90(1.92-4.40)	3.27(2.04-5.26)
	GG	29	4.1	21	3.4	2.91(1.56-5.43)	3.45(1.71-6.96)
Medium	LL	153	21.5	117	18.9	3.07(2.13-4.42)	3.53(2.33-5.35)
	LG	44	6.2	40	6.5	3.65(2.20-6.07)	3.56(2.01-6.30)
	GG	12	1.7	15	2.4	5.02(2.24-11.25)	5.56(2.14-14.44)
High	LL	54	7.6	91	14.7	6.77(4.38-10.44)	8.25(4.97-13.71)
	LG	49	6.9	114	18.4	9.34(6.06-14.40)	10.42(6.29-17.28)
	GG	20	2.8	88	14.2	17.67(10.12-30.85)	18.51(9.70-35.30)

^a ^From the likelihood ratio test comparing the fit of the logistic model that included the main effects of AFB1-DNA adducts levels, genotypes and all potential confounders with a fully parameterized model containing the interaction terms of XPD codon 751 genotypes and AFB1-DNA adducts levels (interact term OR = 1.04, *P*_interaction _= 0.525).

^b ^AFB1-DNA adducts levels were: ≤ 1.00 μmol/mol DNA for low; 1.01 -- 2.00 f μmol/mol DNA for medium; ≥ 2.01 μmol/mol DNA for high.

^c ^Adjusted for age, sex, ethnicity, HBsAg, anti-HCV, and AFB1-exposure years.

## Discussion

To the best of our knowledge, no studies have investigated the role of DNA-repair gene XPD for patients suffering HCC, especially from AFB1 exposure areas. In this study, we analyzed the association between XPD Asp312Asn and Lys751Gln polymorphisms and the risk of HCC among Guangxi population, from a high AFB1-exposure area. The results showed XDP codon 312 polymorphism was not related to HCC risk in Guangxi population. However, we found the genotypes of XPD with codon 751 Gln allele had a substantial association with the increasing risk of HCC (adjusted OR 1.75 for XPD-LG; 2.47 for XPD-GG). Interestingly, a gene-environment interactive effect was found for those populations who had XPD codon 751 Gln alleles, and experienced dissimilar AFB1-exposure years, whose adjusted ORs were 3.70 for XPD-GG and short-term AFB1-exposure years; 18.52 for XPD-GG and medium-term AFB1-exposure years; and 470.25 for XPD-GG and long-term AFB1-exposure years (*P*_interaction _= 0.011). These results may suggest that there were interactions between the genetic polymorphism of XPD codon 751 and AFB1-exposure years, and imply that this polymorphism may have functional significance in HCC induced by AFB1.

HCC is one of major cancer types in Guangxi Zhuang Autonomous Region, China [1,2]. Clinic-epidemiological evidence suggests that the heavy AFB1 exposure via corn and groundnut consumption, main food consumption types for Guangxiese, is the most common cause [1,2]. Our previous studies [6,7] and present study not only supported the aforementioned studies, but also we found the risk of HCC was associated with different AFB1-exposure status. AFB1 is an important chemical carcinogen [3,34], which is mainly metabolized by cytochrome P450 into the genotoxic metabolic AFB1-epoxide that can bind to DNA, causing the formation of AFB1-guanine adducts and inducing DNA damage including base damage and oxidative DNA damage that can be repair by NER pathway [3,4,34].

While XPD protein, encoded by XPD gene, is a DNA-dependent ATPase/helicase that is associated with the TFIIH transcription-factor complex, and plays a role in NER pathway. During NER, XPD participates in the opening of the DNA helix to allow the excision of the DNA fragment containing the damaged base [8,9]. There are four described polymorphisms that induce amino acid changes in the protein: in codons 199 (Ile to Met), 201 (His to Tyr), 312 (Asp to Asn) and 751 (Lys to Gln). The first two are quite rare (~0.04%) in most population, whereas codon 312 and 751 polymorphisms in conserved region of XPD have been extensively studied [8-10]. Several groups have done genotype-phenotype analyses with these two polymorphisms and have shown that the variant allele genotypes are associated with low DNA repair ability [11,12]. For example, Spitz, *et al*. [11] investigated modulation of NER capacity by XPD codon 312 and 751 polymorphisms in lung cancer patients. They found that those individuals carrying with XPD genotypes with codon 312 Asn alleles or/and 751 Gln alleles showed lower NER capacity than those with wild-type genotypes. Rzeszowska-Wolny *et al*. [12] investigated effect of polymorphisms in this gene codon 312 and 751 on DNA damage induced by gamma radiation and its repair in lymphocytes in vitro. They found that XPD 312 Asn alleles were associated with more radiation-induced Damage. Recently, many studies have showed that there is association between these two polymorphisms of XPD and DNA-adducts levels [22,23], p53 gene mutation [14-17], and cancers risk [13,18-30]. These results are controversial; however, most studies have shown that XPD codon 751 Gln alleles and codon 312 Asn alleles are positively related to high cancer risk [11,19,20,25-30]. In this study, we did not found significant evidence for the effects of XPD codon 312 polymorphism on HCC risk, but our data supported that this gene codon 751 Gln alleles increased the risk of AFB1-related HCC. Therefore, the possibility that the XPD protein influences HCC risk through an alternative pathway should not be ignored.

Interestingly, previous several studies have implied that the genotypes of XPD codon 751 be able to interact with environmental risk factors in the process of cancer development [22,27,35]. For example, Terry, *et al*. [22] analyzed the association between XPD codon 751 polymorphism and breast cancer risk. They found this polymorphism may interact with environmental exposures and markers such as polycyclic aromatic hydrocarbon-DNA adducts and cigarette smoking in the breast cancer development. In this study, we found that XPD codon 751 polymorphism would be able to interact with AFB1-exposure years in the process of AFB1-induced HCC. Additionally, we also found some evidence of a joint effects of the genotypes of XPD codon 751 and the levels of AFB1-exposure, although the multiplicative interaction term was not statistically significant.

Those results suggest that this polymorphism may alter the normal protein function, and consequently may be associated with a reduction in DNA repair capacity [8,9]. Thus, the DNA damage induced by AFB1 cannot be repaired effectively and duly, consequently may induce genic mutation such as p53 [14-17], and hepatocellular canceration. Thus, XPD Lys751Gln polymorphism may play a role in the carcinogenetic pathway of Guangxiese HCC.

In this study, we stratified the analysis of XPD codon 751 genotypes by sex. This was done primarily because previous several studies had provided evidence that there might be biological plausibility for gender differences in DNA repair capacity [36-38]. Mayer *et al*. [36] reported a decrease in double-strand break repair in women compared with men. Similarly, Duval *et al*. [37] showed a decrease in mismatch repair capacity and increased microsatellite instability for women compared with men. In addition, findings from Kovtun *et al*. [38] suggest the possibility that X- or Y-encoded factors influence repair or replication of DNA in the embryo. While our study showed the XPD genotypes with codon 751 Gln alleles had higher HCC risk among women than among men (*P*_interaction _< 0.01), which implied a decrease in NER capacity and a increase cancer risk for women compared with men. In support of aforementioned hypothesis, recent several molecular epidemiology researches have reported decreased DNA repair capacity, increased DNA-adduct levels, and increased cancer risk for women compared to men [11,27]. These findings could, therefore, explained increased risk of HCC associated with the XPD genotypes with codon 751 Gln alleles among female population in our study.

## Conclusion

In summary, to the best of our knowledge, this is the first report to investigate association between XPD codon 312 and 751 polymorphisms and the risk of HCC for Guangxi population from an high AFB1-exposure area. We find evidence to suggest that the genotypes of XPD with codon 751 Gln alleles may increase the risk of AFB1-related HCC and the NER pathway may play an important role in the mechanism of action of this genotoxin. However, there were several limitations to our study. Despite XPD polymorphisms analyzed, we did not analyze the genetic polymorphisms of other DNA repair genes involving in the NER such as XPA, XPC, and so on [5]. Our findings were based on relatively small numbers and limited by small number subjects in part of the genotype strata. Therefore, the more genes deserve further elucidation based on a large sample and the combination of genes and AFB1 exposure.

## Abbreviations

AFB1: aflatoxin B1; CI: confidence interval; HBsAg: hepatitis B surface antigen; HBV: hepatic B virus; HCC: hepatocellular carcinoma; HCV: hepatic C virus; OR: odds ratio; NER: nucleotide excision repair; PCR: polymerase chain reaction; RFLP: restriction fragment length polymorphism; XPD: xeroderma pigmentosum complementation group D; XPD-GG: the homozygotes of XPD codon 751 Gln alleles; XPD-LG: the heterozygotes of XPD codon 751 Lys and Gln allele; XPD-LG/GG: the XPD genotypes with codon 751 Gln alleles; XPD-LL: the homozygote of XPD codon 751 Lys alleles.

## Competing interests

The authors declare that they have no competing interests.

## Authors' contributions

YM participated to the design of the study and performed statistical analyses. YFZ participated to analysis and interpretation of data. JGY participated to analysis and interpretation of data. FZB performed the data management. YZH and BCH participated to subjects' collection and performed some screening analyses. All authors read and approved the final manuscript.

## Pre-publication history

The pre-publication history for this paper can be accessed here:

http://www.biomedcentral.com/1471-2407/9/400/prepub
